# Genetic Insight into the Domain Structure and Functions of Dicer-Type Ribonucleases

**DOI:** 10.3390/ijms22020616

**Published:** 2021-01-09

**Authors:** Kinga Ciechanowska, Maria Pokornowska, Anna Kurzyńska-Kokorniak

**Affiliations:** Department of Ribonucleoprotein Biochemistry, Institute of Bioorganic Chemistry Polish Academy of Sciences, Noskowskiego 12/14, 61-704 Poznan, Poland; kciechanowska@ibch.poznan.pl (K.C.); pokornowskamaria@gmail.com (M.P.)

**Keywords:** Dicer, DCL proteins, ribonuclease III, RNase III, RNAi, helicase, DUF283, PAZ, dsRBD, RNA binding proteins

## Abstract

Ribonuclease Dicer belongs to the family of RNase III endoribonucleases, the enzymes that specifically hydrolyze phosphodiester bonds found in double-stranded regions of RNAs. Dicer enzymes are mostly known for their essential role in the biogenesis of small regulatory RNAs. A typical Dicer-type RNase consists of a helicase domain, a domain of unknown function (DUF283), a PAZ (Piwi-Argonaute-Zwille) domain, two RNase III domains, and a double-stranded RNA binding domain; however, the domain composition of Dicers varies among species. Dicer and its homologues developed only in eukaryotes; nevertheless, the two enzymatic domains of Dicer, helicase and RNase III, display high sequence similarity to their prokaryotic orthologs. Evolutionary studies indicate that a combination of the helicase and RNase III domains in a single protein is a eukaryotic signature and is supposed to be one of the critical events that triggered the consolidation of the eukaryotic RNA interference. In this review, we provide the genetic insight into the domain organization and structure of Dicer proteins found in vertebrate and invertebrate animals, plants and fungi. We also discuss, in the context of the individual domains, domain deletion variants and partner proteins, a variety of Dicers’ functions not only related to small RNA biogenesis pathways.

## 1. Introduction

The gene encoding for Dicer ribonuclease, as reported by Hansen et al. [1], was first revealed in 1994 by Bass et al. in a screen of the *Xenopus* ovary cDNA expression library for double-stranded RNA (dsRNA) binding proteins (dsRBPs) [2]. Later, in 1999, Provost et al. published the first literature record related to human Dicer (hDicer) [3]. The authors isolated hDicer cDNA clones from a yeast two-hybrid screen, using as a bait, 5-lipoxygenase, a pivotal protein in cellular leukotriene synthesis. Analysis of the predicted amino acid sequence of the newly identified protein revealed its high homology to a hypothetical helicase K12H4.8 from *Caenorhabditis elegans* as well as the presence of a ribonuclease III (RNase III) signature motif and a dsRNA binding domain (dsRBD) [3]. A year later, in 2000, a detailed description of the *DICER1* gene was published [4], at that time under the name *“HERNA”* (helicase with RNase motif), because the protein product of the gene contained conservative evolutionary motifs characteristic of the ATP-dependent RNA helicase, i.e., ATP binding motif and DEXD/H-box (Asp-Glu-X-Asp/His), and RNA binding motifs [4]. Expression of *HERNA* was shown to be ubiquitous and tissue-specific [4]. The name “Dicer” was first introduced into the scientific world in 2001 by Bernstein, Caudy, Hammond and Hannon [5], and it derived from the ability of the enzyme to “dice”, i.e., digest dsRNA into uniformly sized small RNAs, ~22 nucleotides (nt) in length. As Dicer displayed specificity for dsRNAs, it was assigned to the family of RNase III endoribonucleases [5].

Relating to the plant kingdom, the first literature record of Dicer goes back to 1999, when Jacobsen et al. identified in *Arabidopsis thaliana* the *CARPEL FACTORY (CAF)* gene, which played a role in floral meristem determinacy [6]. The *CAF* gene encoded a putative protein of 1909 amino acids containing an amino (N)-terminal DExH/DEAD-box type RNA helicase domain and a carboxy (C)-terminal RNaseIII-like domain followed by a dsRNA binding domain [6]. In those days, as mentioned above, a highly similar protein of an unknown function was found to be encoded by a fungal and an animal genome, indicating that the CAF-type proteins could play a role in many eukaryotic organisms. Moreover, the structure of CAF protein suggested that it could act as an RNA processing enzyme. Later, in 2002, Reinhart et al. made the observation that the plant microRNA (miRNA) accumulation is dependent on the CAF protein [7]. Nowadays, the *CAF* gene is referred to as *DICER-LIKE1* (*DCL1*), which encodes well known endonuclease that produces plant miRNAs [8]. Identification of three other genes similar to *CAF* in *A. thaliana* led to the recognition of additional DICER family members: *DCL2*, *DCL3* and *DCL4* [9].

**Figure 1:**
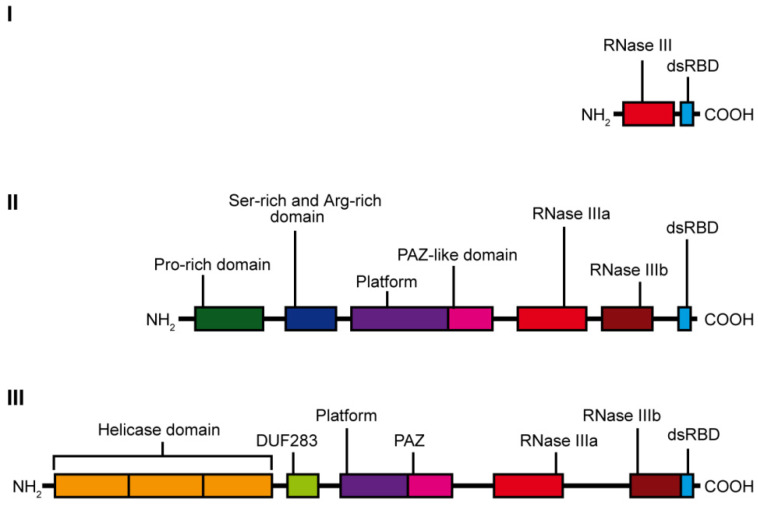
Scheme showing the domain organization of the individual classes of the RNase III family enzymes: class I—represented by *E. coli* ribonuclease III, class II—represented by human Drosha ribonuclease and class III—represented by hDicer ribonuclease.

RNase III specifically hydrolyzes phosphodiester bonds found in double-stranded regions of RNAs and generate products having 5′-phosphate, 3′-hydroxyl, and 2-nt 3′-overhangs [10]. Based on the domain organization and biological functions, the RNase III family can be divided into three subfamilies (Figure 1). The first subfamily includes bacterial RNase III and yeast Rnt1, which contain only one RNase III domain and one dsRBD domain. RNase IIIs of this subfamily form homodimers to assemble a dsRNA-cleavage center [11]. The bacterial RNase III and yeast Rnt1 are essential for processing of ribosomal RNA precursors [12]. Moreover, Rnt1 functions in the maturation of both small nuclear RNAs [13,14] and small nucleolar RNAs [15], in processing of mRNA [16], and mRNA quality control [17]. The second subfamily of RNase III is represented by enzymes containing two RNase III domains and a single dsRBD in the C-terminal region, e.g., ribonuclease Drosha. Drosha is the core nuclease initiating processing of a primary miRNA precursor (pri-miRNA) in the nucleus. Products of Drosha cleavage are 50–70-nt pre-miRNAs with hairpin structures [18]. Drosha is also involved in mRNA processing [19] and, presumably, in ribosomal RNA maturation [20,21]. Finally, the third subfamily of RNase III comprises Dicer-type enzymes. Dicer-type RNases consist of two RNase III domains and a dsRBD in the C-terminal region, and they also include helicase domain, a DUF283 (Domain of Unknown Function) and a PAZ (Piwi–Argonaute–Zwille) domain in the N-terminal region. Nevertheless, there are a few examples of Dicers that lack some of these domains, e.g., the Dicer from *Giardia intestinalis*, which does not possess helicase, DUF283, and dsRBD domains [10], *A. thaliana* DICER-LIKE 3 (DCL3) protein, which lacks a DUF283 domain [22], or the green alga *Chlamydomonas reinhardtii* DCL3, which lacks the PAZ domain [23]. The second and third subfamily members use intramolecular dimerization of their two RNase III domains to form a cleavage center. Dicer ribonucleases are mostly known for their essential role in the biogenesis of small regulatory RNAs, such as miRNAs or small interfering RNAs (siRNAs). They recognize and cleave pre-miRNAs or dsRNAs into functional 21–23-nt miRNAs or siRNAs, respectively. In this manuscript, we review the information on the domain organization and structure of Dicer proteins found in vertebrates, with specific emphasizes on hDicer, invertebrates, plants and fungi. In relation to the individual Dicer’s domains, we follow the miRNA and siRNA pathways, and summarize recently revealed roles of Dicers in many aspects of cellular life.

## 2. Evolution of Dicer-Type Proteins

Evolutionary studies indicate that Dicer and its homologues developed only in eukaryotes, and the Dicer gene underwent duplication during the emergence of the first multicellular organisms [24]. Then, Dicer genes differentiated independently in the animal, plant and fungal kingdoms [24]. Animals developed two genes coding for Dicer proteins, Dicer-1 and Dicer-2. Dicer-1 is responsible for pre-miRNA recognition and miRNA production, while Dicer-2 cleaves dsRNA to generate siRNA [25]. One of the best characterized Dicer-2 proteins in the animal kingdom is the insect Dicer-2 which, by cleaving viral dsRNAs, plays an important role in the response to viral infections [26,27]. Dicer-2 was subsequently lost from lineages that developed complex immune systems, such as vertebrates [24]. Moreover, the Dicer gene was lost from some protozoan parasites and some fungi, e.g., the model organism *Saccharomyces cerevisiae* and other closely related yeasts [28,29]. In the plant kingdom, at least four genes encoding Dicer-type enzymes developed [24]. Plant Dicers are termed DICER-LIKE (DCL) proteins. DCL1 is involved in the production of miRNAs, typically 21-nt long, from primary transcripts that contain stem-loop structures, while DCL2, DCL3 and DCL4 are involved in siRNA production [30]. Precisely, DCL2 produces 22-nt siRNAs which can silence transgenes in the plant response to viral infections, and similarly to its insect homolog Dicer-2, is a part of the antiviral defense system [31,32]. DCL3 generates 24-nt siRNA, which participates in chromatin modifications, thus influencing the chromatin structure [33]. DCL4 is responsible for the formation of trans-acting siRNAs (tasiRNAs) [34]. DCL4 is also involved in the antiviral response [31]. Some plants encode more than four DCL proteins; for example, in *Medicago truncatula*, six DICER-LIKE protein genes are expressed: *DCL1*, *DCL2*, *DCL3*, *DCL4* and two *DCL2* homologues, including the variant that encodes a truncated version of the DCL2 protein [35]. Plant DCL proteins form a protein family, whose diversification time dates to the emergence of mosses (*Physcomitrella patens*) [36]. DCL proteins are ubiquitously but not evenly expressed in plant tissues, depending on developmental stages and response to stresses [36].

It is also important to mention DICER proteins encoded by algae. *Chlamydomonas reinhardtii* is a single-cell green alga, which has a complex RNA silencing machinery involving three DCL proteins (DCL1-3) and three Argonaute proteins (AGO1-3) [37,38]. The miRNAs silencing system in algae is distinct from that of land plants and even may have common features with the miRNA silencing mechanism found in animals. DCL3 of *C. reinhardtii* is known to process the majority of miRNA, although it lacks the PAZ domain [23]. Another unique feature of DCL3 is a proline-rich domain on the N-terminal side of the RNase III motifs, though a similar domain is also present in a related protein, a ribonuclease Drosha that also lacks the PAZ domain [23,39]. Considering these features, it could be possible that *C. reinhardtii* DCL3 performs the function of Dicer and Drosha, being involved in several stages of miRNA biogenesis [23]. Plant and animal miRNA pathways are very different, thus it is likely that they evolved separately from an ancestral RNA silencing pathway with Dicer proteins [40,41]. The algal miRNA pathway is also distinct from that of higher plants, and the two evolutionary scenarios could explain those differences. The first scenario assumes that animal, higher plant, and algal miRNA pathways all evolved independently of each other. A second scenario takes into account that an animal-like miRNA pathway evolved early and persisted in lower plant lineages, including the green algae, though it was not retained in higher plants. The second scenario is more likely because *C. reinhardtii* and animal miRNA pathways share the presence of a Drosha-like structure (i.e., the presence of the proline-rich domain and the absence of the PAZ domain) of the miRNA processing enzyme [23].

Although evolutionary studies indicate that the ribonuclease Dicer is absent in bacteria and archaea, the helicase domain of Dicer displays high sequence similarity to its prokaryotic orthologues, archaeal Hef proteins that belong to Superfamily 2 (SF2) helicases [42,43]; the RNase III domains of Dicer are highly similar to bacterial RNase III [44]. A combination of the SF2 helicase and RNAse III domains in a single protein is considered a eukaryotic signature, and is supposed to be one of the critical, early events that lead to the consolidation of the eukaryotic RNA interference (RNAi) system [44].

## 3. Dicer Structure and the Importance of Its Domains

**Figure 2:**
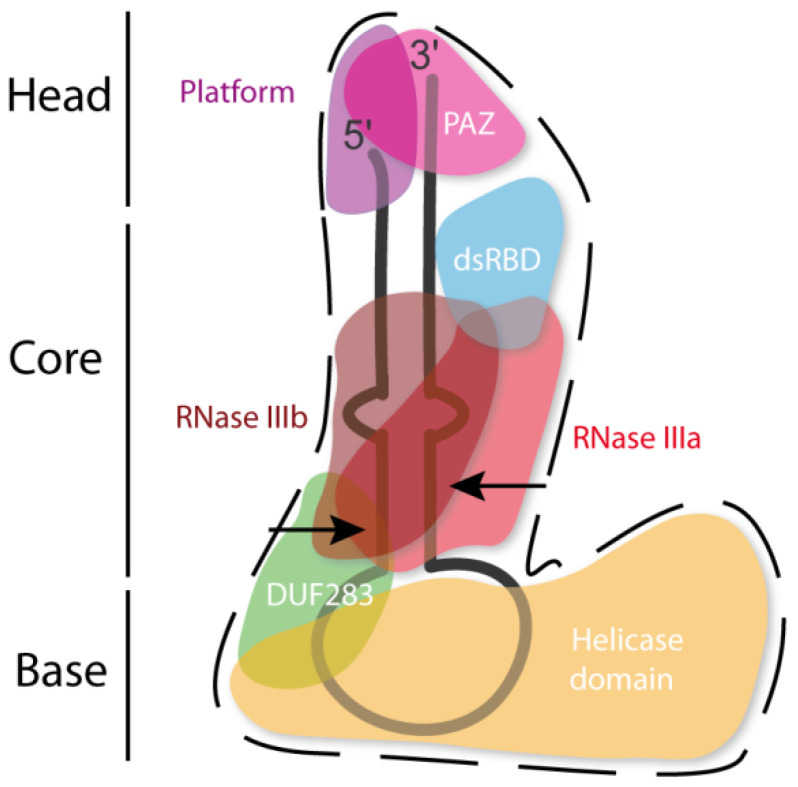
Scheme of the tertiary structure of hDicer in complex with pre-miRNA, prepared based on Liu et al. [49]. Arrows indicate the pre-miRNA cleavage sites by RNase III domains.

**Figure 3:**
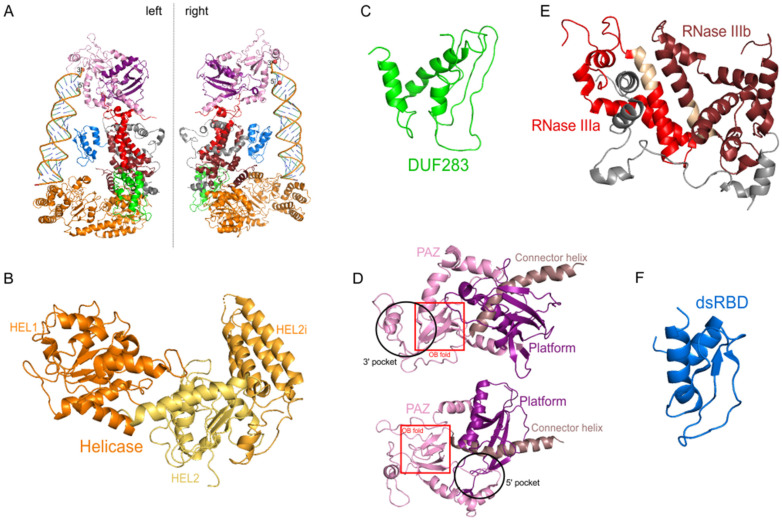
The 3D structure of hDicer (PDB entry 5ZAL) and hDicer individual domains visualized by PyMOL. (**A**) hDicer in complex with pre-let-7 (the loop region is not seen in this structure), the 5′-phosphate and the 3′-hydroxyl are indicated with spheres; (**B**) the helicase domain, with its three internal subdomains, is indicated in orange and yellow; (**C**) the DUF283 domain is indicated in green; (**D**) the Platform–PAZ–Connector helix cassette is shown in two orientations; the upper panel presents the 3′ pocket, whereas the lower panel exposes the 5′ pocket. The Platform domain is indicated in purple, PAZ in light pink, and Connector helix in brown. The OB fold within the PAZ domain is marked by the red rectangle; (**E**) the catalytic core—RNase IIIa and IIIb domains are indicated in red and wine red, respectively; fragments connecting RNase III domains are indicated in gray; fragments constituting the RNase III signature motifs are colored in beige; (**F**) the dsRBD domain is indicated in blue.

The hDicer ribonuclease is encoded by the *DICER1* gene located on chromosome 14 in the subtelomeric region 14q32.13. The *DICER1* gene spans a region of about 72 kbp and contains 29 exons. *DICER1* is considered a housekeeping gene [45], although, compared to other housekeeping genes, it has an unusually long 3′-untranslated region (3′-UTR), i.e., over 4000 base pairs (bp) [45]. The protein product of *DICER1* consists of 1922 amino acids (~220 kDa), and similar to other members of the third subfamily of RNases III, comprises a putative helicase domain, DUF283, PAZ, two RNase III domains (RNase IIIa and RNase IIIb) and dsRBD (Figure 1) [11]. Structural studies on hDicer have revealed new functional domains adjacent to the PAZ domain, namely, Platform and Connector helix [46,47]; the Platform-PAZ-Connector helix fragment is often referred to as “hDicer PAZ cassette” [47] or “PPC cassette” [48]. The experiments conducted with the use of cryo-electron microscopy (cryo-EM) techniques, revealed that the three-dimensional structure of the hDicer resembles the letter L [46,49]. Within this structure, the head, core and base can be distinguished. The head constitutes the PAZ and the Platform domains, the RNase III domains are in the core, while the helicase domain forms the base (Figure 2 and Figure 3A) [49].

### 3.1. The Helicase Domain

The helicase domain is considered to be one of the most conserved regions among all Dicer proteins [50], and shows a high sequence similarity to the helicases of the superfamily SF2 [42,43,44]. SF2 is the largest and most diverse of the helicase superfamilies. Based on sequence homology, SF2 was divided into a number of different families including, but not limited to, RIG-I-like helicases, DEAD-box helicases or type I restriction enzymes [51]. The hDicer’s helicase comprises three subdomains termed HEL1, HEL2i, and HEL2 [50], which are structurally organized into three lobes (Figure 3B) [49]. HEL1 (containing the DExD/H-box motif) and HEL2 (termed also helicase C) are conserved for all helicases from the DExD/H and the RIG-I-like families of SF2 helicases [51,52], while the HEL2i domain is conserved only for the RIG-I-like family.

In general, the DExD/H-box helicases are capable of unwinding RNA or DNA duplexes, while RIG-I-like helicases can translocate along nucleic acids [51]. Both these activities are fueled by hydrolysis of ATP [53]. However, the helicases of vertebrate Dicers containing the DExD/H-box motif have never been found to be an ATP-dependent. Furthermore, *D. melanogaster* Dicer-1, which is responsible for generating miRNAs, contains a degenerated HEL1 domain unable to hydrolyze ATP [25], and *Giardia* Dicer has no helicase domain [10]. In contrast, there are numerous examples of Dicer’s helicase domains that have a functional DExD/H-box motif and require ATP for their activities; such helicases are found in, e.g., *D. melanogaster* Dicer-2 and *C. elegans* Dicer [54], plant DCL proteins [35,55,56] and fission yeast Dicer [57]. Results presented by Kidwell et al. suggest that the role of the helicase domain may be conserved from fungi to humans [50]. The thermophilic fungus *Sporotrichum thermophile* genome encodes two Dicer proteins; Dicer-1 cleaves pre-miRNAs, whereas Dicer-2 processes dsRNAs. Similar to *D. melanogaster* Dicer-2 [58], only *S. thermophile* Dicer-2 has ATPase activity. Importantly, in plant DCL1 proteins, the ATPase function of the helicase domain has been retained, presumably due to the structure of plant miRNA precursors that have long but imperfect stems of the hairpin structure. It was observed that the deletion mutant of *Arabidopsis* DCL1 lacking the helicase domain showed higher processing activity in vitro and was no longer dependent on ATP [55]. Moreover, in vitro, the DCL1 helicase domain was shown to be required for precise processing of some pre-miRNAs [55].

Functional RIG-I-helicase-type subdomains, i.e., HEL1, HEL2i, and HEL2, are present in, e.g., insect Dicer-2 and plant DCL proteins. In these enzymes, the helicase domain was shown to act as an RNA virus infection sensor that activates the appropriate effector RNAi pathway and, consequently, triggers the cleavage of viral RNA [59]. In vertebrates, a separate protein, the RIG-I helicase, serves as a receptor for the innate immune system, which is a part of the first line of defense against RNA viruses. The helicase domain of RIG-I plays an important role in recognition and binding of viral RNAs, and in coupling RNA binding to antiviral signaling [60]. Once stimulated by the viral dsRNA, RIG-I activates the signaling pathways of transcription factors triggering the production of interferon-β and activation of many genes involved in the mobilization of the immune system [61]. It is assumed that vertebrate Dicers lost the ability to recognize viral RNAs by their helicase domains due to development of other intracellular RNA virus sensors, e.g., RIG-I helicases [1]. Consequently, the helicase domains of vertebrate and invertebrate Dicers might develop different activities [1].

In organisms that have only one Dicer, e.g., mammals and nematodes, the helicase domain discriminates between pre-miRNA and pre-siRNA substrates [62,63], but the discrimination mechanism seems to be different for mammalian and nematode Dicers. It is known that the hDicer’s helicase domain, by interacting with the apical loop of pre-miRNA substrates, can differentiate pre-miRNAs from pre-siRNAs [64,65]. In addition, hDicer cleaves pre-miRNAs much more efficiently than pre-siRNAs, and the difference in cleavage rates between these substrates is attributed to the presence of the helicase domain [62]. The hDicer’s helicase plays also an important function in the processing of thermodynamically unstable hairpin structures [66]. Moreover, it was demonstrated that the helicase domain deletion enhanced hDicer’s ability to process pre-siRNAs [67]. Referring to nematodes, mutations affecting ATP hydrolysis in the helicase domain of *C. elegans* Dicer were shown to reduce the amount of certain endogenous siRNAs, but the same mutations did not affect miRNA generation as well as the exogenous pre-siRNAs processing [68]. Nevertheless, most invertebrates segregate processing of RNA substrates between two Dicer proteins, Dicer-1 and Dicer-2 [24,25]. In addition, it was demonstrated that invertebrate Dicers cleave pre-siRNA substrates differently, depending on substrate termini, i.e., dsRNAs with blunt termini are progressively cleaved in an ATP-dependent way, while substrates with 3ʹ-overhanging termini undergo distributive cleavage that does not require ATP [50,54]. Importantly, a cryo-EM structure of *Drosophila* Dicer-2 in complex with a blunt dsRNA, revealed that a blunt-ended dsRNA substrate binds to the helicase domain [69], while substrates with 2-nt 3ʹ-overhanging termini are bound by the PPC cassette of Dicer [47].

The helicase domain of Dicer proteins can also serve as a platform for binding of dsRBPs, e.g., hDicer can assemble with the TAR RNA-binding protein (TRBP) or protein activator of protein kinase R (PACT) [1,27]. TRBP and PACT are important regulators that contribute to substrate binding during small regulatory RNA production [70]. Binding of these two dsRBPs to the hDicer’s helicase domain was shown to be mutually exclusive [71]. It was shown that Dicer, in association with PACT, preferentially cleaves pre-miRNAs rather than pre-siRNAs. This discrimination is less prominent when Dicer is in complex with TRBP [70]. The direct binding of TRBP to pre-miRNA substrates was proposed to increase the initial rate of substrate recognition by Dicer [63]. Moreover, TRBP binding was shown to increase the stability of Dicer/substrate complexes and the efficiency of substrate cleavage [63]. TRBP and PACT not only influence substrate discrimination by Dicer but also may change the cleavage site selection, thereby triggering the generation of different sized miRNAs (iso-miRs) [71,72,73]. Importantly, an alternate pre-miRNA processing can have a significant change on the targeted mRNA cleavage or on the translational repression target. Moreover, TRBP acts as a bridge between Dicer and Argonaute (Ago) proteins during the RNA-induced silencing complex (RISC) assembly, and presumably has a role in guide strand selection within the miRNA/siRNA duplexes [62,71,74].

In other species, the Dicer’s helicase domain interacts with a variety of dsRBPs displaying similar functions to human TRBP or PACT. For example, the Loquacious (Loqs) and R2D2 proteins are interactors of *Drosophila* Dicer-1 and Dicer-2 proteins [1,75,76,77]. Loqs and R2D2 were found to influence differently small regulatory RNA biogenesis [72,78,79]. Moreover, Loqs isoforms were demonstrated to tune the length of mature miRNAs [72].

### 3.2. The DUF283 Domain

The name “DUF” is given to the protein domains that have no characterized functions and is followed by the number assigned to a particular domain. The DUF283 domain of Dicer proteins shows high structural similarity to typical dsRBDs [80] that can be found in eukaryotic, prokaryotic and viral proteins [81]. Nevertheless, the role of DUF283 in the small RNA biogenesis pathway is still elusive.

DUF283 was initially suggested to play a significant role in the pre-miRNA processing as hDicer variants lacking both the DUF283 and helicase domains lost the cleavage activity [82]. Deletion of the DUF283 and helicase domains also affected pre-miRNA processing by *D. melanogaster* Dicer-1 [83]. Later, it was shown that the deletion of the DUF283 domain itself increases binding but reduces cleavage efficiency of dsRNA by hDicer. However, the DUF283 domain deletion variant of hDicer can bind and cut pre-miRNA substrates with similar efficiency as the wild-type enzyme [62]. The structure of DUF283 from *A. thaliana* DCL4, examined using nuclear magnetic resonance (NMR) spectroscopy, showed that DUF283 adopts an αβββα structural motif characteristic for the canonical dsRBDs [80]. A similar dsRNA-binding motif was predicted [84], and then experimentally confirmed (Figure 3C) [49], for the hDicer DUF283 domain. Nevertheless, experimental studies have revealed only trace dsRNA-binding activity of the DUF283 domain from *A. thaliana* DCL4 [80], and have never revealed even weak dsRNA-binding activity for DUF283 of hDicer [85]. It was found, however, that the hDicer DUF283 domain can bind single-stranded nucleic acids in vitro [85]. Moreover, it was shown that DUF283, by supporting the base pairing of complementary sequences found in nucleic acids, can perform a function characteristic of nucleic acid chaperone-like proteins or nucleic acid annealers [85]. In addition, presumably due to the presence of the DUF283 domain, hDicer can support hybridization between a small RNA and a complementary sequence of a longer RNA, even when both complementary sequences are trapped within stable secondary structures [86]. Putatively, hDicer relaxes local secondary structures via transient interactions with single-stranded regions of RNA. Therefore, it is believed that hDicer, by remodeling RNA structures and supporting RISC in targeting complementary sequences located within stable secondary structures of mRNA transcripts, might be directly involved in translational control of gene expression [86].

The DUF283 domain is often referred to as a “dimerization domain”, because it was found to be involved in the binding of Dicer partner proteins. The DUF283 domains from *A. thaliana* DCL1 and DCL4 were shown to bind HYL1 (hyponastic leaves 1) and DRB4 (dsRNA-binding protein 4), respectively [80]. Moreover, the DUF283 domain of hDicer binds the adenosine deaminase acting on RNA 1 (ADAR1) protein, which stimulates both the efficiency of pre-miRNA cleavage by Dicer and the efficiency of miRNA transfer to Ago [87].

### 3.3. Functional Core of DICER: The PAZ and Two RNase III Domains

*Giardia* Dicer, called a minimal Dicer, comprises the PAZ and tandem RNase III domains, and lacks many of the domains and regions characteristic of Dicers in higher eukaryotes [10]. Therefore, the PAZ and two RNase III domains are considered as a functional core of the canonical Dicer-type enzymes. The PAZ domain of Dicer enzymes shows high structural similarity to the PAZ domains found in other proteins involved in small RNA processing and the effector stages of RNAi [88]. In general, the PAZ domain can be found in two protein families: the PAZ-Piwi Domain Proteins family (PPD) and the Dicer family [89]. Well known PPD proteins are Ago proteins. Structural studies revealed that PAZ domains contain a variant of the OB (Oligonucleotide/Oligosaccharide-Binding) fold, a module that often binds single-stranded nucleic acids (Figure 3D). Precisely, the crystal structure of the PAZ domain of *D. melanogaster* Ago2 demonstrated that this domain is responsible for binding of the 3′-ends of single-stranded regions of RNA [90,91]. Likewise, crystal structures of the PAZ domain from human Ago1 bound to siRNA-like duplex showed that PAZ anchors the 2-nt 3′-overhang of the siRNA-like duplex within a highly conserved binding pocket [92]. Interestingly, very recently it was found that sulfonamide antibiotics, by binding to human Ago2 PAZ domain, can inhibit RNAi [93].

Referring to the mechanism of Dicer action, the initial recognition and anchoring of the substrate take place within the region spanning the Platform–PAZ–Connector helix cassette (Figure 3A,D) [47]. This region contains two adjacent pockets, a 2-nt 3′-overhang-binding pocket (3′ pocket) located within the PAZ domain, and a phosphate-binding pocket (5′ pocket) positioned within the Platform and PAZ domains [47]. The 3′ pocket and the 5′ pocket are situated within close proximity to ensure simultaneous accommodation of the respective substrate ends [94]. Pre-miRNAs, compared to pre-siRNAs, usually have only partially paired 5′- and 3′-ends, which are relatively easy to split up [47]. Consequently, 5′-ends of pre-miRNA substrates can be easily anchored in the 5′ pocket. Importantly, it was found that the 5′ pocket is highly conserved among most Dicer homologues producing miRNAs, and it is missing in Dicer from lower eukaryotes that lack the miRNA pathway, such as fungi and *Giardia* [94]. Notably, the 5′ pocket as well as the whole PAZ domain is missing in DCL3 from *C. reinhardtii*, which is able to produce miRNAs [23], while the plant enzyme producing miRNA (DCL1) is only partially conserved in the region spanning the 5′ pocket [94]. Interestingly, *Drosophila* Dicer-2 has its unique 5′ pocket, which was demonstrated to be important for the fidelity of 21-nt long siRNA production, but not for the efficiency of the performed cleavage [95].

The PAZ domain has been initially suggested to mediate complex formation between the proteins from PPD and Dicer families [88]. Later, it was shown that the Dicer RNase III domain is required for binding to Ago [88,96,97]. By interacting with the RNase III domain of Dicer, Ago proteins are brought to the close proximity of the RNA cleavage product of Dicer (miRNA or siRNA). Ago2, bound to miRNA or siRNA, form RISC which mediates gene silencing by targeting messenger RNA. Interestingly, in vitro studies have suggested that interactions between hDicer and human Ago2 may block the cleavage activity of hDicer [88]. Considering both the presence of hDicer in RISC [21] and the proposed direct role of hDicer in the post-translational control of gene expression [86], indeed the cleavage activity seems to be dispensable for hDicer when targeting transcripts to expose miRNA target sites for RISC.

The simplest Dicer-type proteins, such as Dicer-like1 protein found in the early diverging protozoan *Trypanosoma brucei*, contain just two RNase III domains [98]. Although the budding yeast *S. cerevisiae* does not have Dicer and an active RNAi pathway, other yeasts: *Saccharomyces castellii* and *Candida albicans* [99], and *Kluyveromyces polysporus* [100], do have simple Dicer proteins composed of only a single RNase III domain and a dsRBD domain. Likewise, Dicer of another protozoan parasite Entamoeba histolytica comprises a single RNase III domain, but it lacks a typical dsRBD [101]. A common feature of all these so-called “noncanonical Dicers” is a lack of the PAZ domain. Biochemical analyses showed that noncanonical Dicers, as dimers, bind cooperatively along the dsRNA substrate, and the distance between consecutive active sites determines the length of the generated products, i.e., ~23-nt RNAs [100]. In the case of canonical Dicer enzymes, the length of the produced miRNAs or siRNAs is determined by the distance from the PAZ domain to the RNase III domains (Figure 3A) [46,49,102]. Additionally, spatial orientation of the Platform and PAZ relative to the RNase III domains seems to be important for measuring dsRNA of the defined length [103]. It was demonstrated that, in contrast to the PAZ-containing Dicers, which can bind the substrate termini, noncanonical Dicers initiate processing in the interior and work outward [100]. Moreover, in vitro studies revealed that the PAZ domain deletion variants of hDicer do not process pre-miRNAs and pre-siRNAs; however, they can cleave single-stranded RNA and DNA substrates, and the resulting products differ in size from typical hDicer products [104]. These results suggest that when the PAZ domain is absent (or not available), other hDicer domains may contribute to substrate binding, and in such cases, noncanonical products can be generated. The above results are supported by earlier findings demonstrating that hDicer, when interacting with other proteins, can generate noncanonical products (~55-nt and ~10–12-nt RNAs) from a pre-miRNA substrate [105]. In the aforementioned case, the interacting protein was 5-lipooxygenase, one of the enzymes involved in the biosynthesis of inflammatory messengers. Association of both these proteins was supposed to influence the structure of hDicer, which might affect hDicer cleavage specificity [105]. Interestingly, RNAs of the length of ~55-nt and ~10–12-nt were also produced from a pre-miRNA substrate when the PAZ deletion variant of hDicer, composed of only the RNase IIIb and dsRBD domains, was used in the in vitro cleavage studies [105]. The ~10–12-nt RNAs are also produced by *E. coli* RNAse III comprising one RNase III domain and one dsRBD domain [106].

The pattern of recognition and cleavage by plant DCL proteins differ from the mechanisms observed for animal Dicers. The plant miRNA precursors are much more variable in size and shape than the animal miRNA precursors and, importantly, they are completely processed in the nucleus by DCL1 [41,107,108]. The plant miRNA precursors are processed in two different ways. In the first scenario, the miRNA precursor is bound ~15–17-nt below the lower stem, which determines the position of the first cut by DCL1; the second cut occurs 21-nt away from the first one, releasing the miRNA duplex [109,110,111]. This type of processing is named “base-to-loop” and resembles the processing of animal pre-miRNAs [112]. In the second scenario, the miRNA precursor processing starts from the cleavage below the terminal loop, and further processing continues toward the base of the precursor [111,113]. This type of processing is called “loop-to-base” [113].

RNase III enzymes are highly conserved in the Bacteria and Eukarya, however, are only sporadically observed in the Archaea [114]. Simple RNase III comprising a single endonuclease domain presumably appeared in an early bacterium, and then entered the eukaryotic lineage, e.g., as an endosymbiont [114]. The subsequent appearance of two RNase III domains within one protein was probably caused by duplication of the RNase III coding gene [115]. Then, these RNase III domains diversified, giving rise to RNase IIIa and IIIb domains found in the second and third subfamily of RNases III. RNase IIIa and IIIb domains form an intramolecular dimer and constitute the catalytic core of the canonical Dicer-type enzymes (Figure 3E) [11]. The sequence of the RNase III domain is characterized by a ~10 amino acid stretch of conserved residues, which is known as the RNase III signature motif (Figure 3E). This motif is crucial for binding divalent metal ions (preferably Mg^2+^) important for catalytic activity, or stabilization the dimeric structure of the enzyme [10,116,117]. Nevertheless, magnesium cations can be substituted by manganese, nickel or cobalt, which results in an altered cleavage specificity of RNase III enzymes [115]. Importantly, recognition and binding of RNA substrates are possible in the absence of divalent metal ions, and this characteristic is useful when studying RNase III-substrate interactions [118,119].

The mechanisms of substrate recognition and cleavage by RNase III orthologs have been provided by studies on *E. coli* RNase III [11,120]. Precisely, each RNase III domain independently catalyzes the hydrolysis of a phosphodiester bond within one strand of a double-stranded substrate. In the case of Dicer-type enzymes and pre-miRNA substrates, the RNase IIIa domain cleaves the pre-miRNA 3′ arm that contains the 3′-overhangs, whereas the RNase IIIb domain cleaves the arm that contains the 5′ phosphate [11].

### 3.4. The dsRBD Domain

The dsRBD domain is a small conserved protein domain found in eukaryotic, prokaryotic and viral proteins (Figure 3F); probably dsRBDs were even present in the last common ancestor of metazoans [121]. The dsRBD domain is composed of 65–70 amino acids adopting an αβββα fold [122], similar to the noncanonical dsRNA-binding motif with an αβββα fold of the DUF283 domain (Figure 3C) [122]. This type of the protein fold is considered as a rather rigid fold that does not show substantial structural changes upon dsRNA binding, although parts of the fold display a high degree of mobility, at least when not interacting with RNA [115]. Although the dsRBD-containing proteins can bind to very specific dsRNA targets, the binding of dsRBDs to dsRNA is commonly believed to be shape-dependent rather than sequence-specific. Some dsRBD-containing proteins recognize and bind the substrate bearing the A-form RNA helix, while others prefer stem-loop structures within RNA [123,124,125]. Discrimination between DNA and RNA duplexes relies on interactions between the dsRBD-containing protein and the 2′-OH moiety of ribose.

The central property of dsRBD is to bind to dsRNA, and proteins that include this domain were found to be involved in many aspects of cellular life, including RNA editing, RNA processing, RNA metabolism, RNA transport, RNA silencing, and even antiviral response [81]. Originally it was suggested that the dsRBD of hDicer plays only an auxiliary role in binding to RNA substrates [64]. However, later it was shown that the presence of dsRBD is necessary for substrate binding when hDicer lacks the PAZ domain [64]. A simple RNase III enzyme containing only the RNase III and dsRBD domains, e.g., *E. coli* RNase III, form a homodimer to generate ~11-bp products from dsRNAs [106]. The proposed catalytic pathway for RNase III enzymes assumes that dsRBD plays an important function by engaging the dsRNA substrate and handing it over to the dimeric active site composed of the RNase III domains. After cleavage, dsRBDs take the product over and release it, making the enzyme ready for another round of cleavage [1,114]. Interestingly, it was shown that the C-terminal fragment of hDicer, composed of two RNase III domains and dsRBD, cleaves a dsRNA substrate into ~15-bp products [64]. The addiction of the N-terminal fragment, containing the DUF283 and the PAZ domains, to the C-terminal fragment of hDicer restored production of the characteristic 22-bp products by the reconstituted hDicer [64].

Mammalian Dicers predominantly localize to the cytoplasm to perform pre-miRNA cleavage, but several reports have provided evidence for nuclear function and localization of these proteins [126,127]. The shuttling properties of hDicer has been attributed to the nuclear localization signal (NLS) comprising a cluster of positively charged residues on the surface of the folded dsRBD [128,129]. The dsRBD-dependent import of hDicer is mediated by Importin-β, Importin-7 and Importin-8 [128]. However, it was found that the NLS can be masked by other Dicer’s domains (e.g., the helicase domain [128]) or associating RNA molecules [130]. Similar to hDicer, the C-terminal dsRBD of the yeast *Schizosaccharomyces pombe* Dicer is also critical for the nuclear localization of the protein. In contrast to hDicer, *S. pombe* Dicer localizes predominantly in the nucleus, near the nuclear pore complexes [129]. It was shown that the dsRBD from *S. pombe* Dicer has a C-terminal extension composed of an α-helical turn followed by a zinc-coordination motif [129]. This structure makes a protein–protein interaction surface for a yet unidentified nuclear protein binding that may cause the nuclear retention of the *S. pombe* Dicer. Interestingly, the extended dsRBD of the *S. pombe* Dicer is a temperature sensitive domain which acts as a thermo-switch. When the temperature rises above 34 °C, the nuclear retention properties of the dsRBD changes, and the *S. pombe* Dicer moves to the cytoplasm, with important consequence for stress-dependent gene regulation and response [131].

## 4. A Variety of Dicer Functions

In addition to miRNA and siRNA production, Dicer has been implicated in the biogenesis of a group of small RNAs, which are derived from tRNAs, called tRNA-derived fragments (tRFs) [132]. Studies on *A. thaliana* suggest that DCL proteins could be involved in tRF biogenesis in plants [133]. However, similar data from *Drosophila* and *S. pombe* revealed that the lack of Dicer does not significantly affect tRF levels [134]. Some tRNAs can fold into structures that resemble canonical Dicer substrates, thus it is possible that Dicer-dependent tRFs processing has evolved in certain organisms as a mechanism with biological relevance [130].

Beyond the biogenesis of small regulatory RNAs, Dicer also performs functions that are related to its nuclear localization. In organisms such as flies, plants and fission yeast, nuclear Dicer is involved in formation of heterochromatin [135]. In addition, it was demonstrated that, in mouse embryonic stem cells, chromatin modifications and DNA methylation of centromeric repeats are affected upon Dicer knockout, leading to accumulation of RNAs transcribed from centromeric repeats [136]. More recently, it was shown that, in mouse germ cells, Dicer is necessary for normal spermatogenesis, since during this process it controls pericentric heterochromatin expression [127]. Additionally, it was demonstrated in mice that Dicer is highly expressed in developing cerebrum, and lack of this protein causes the loss of cerebellar progenitor cells, presumably due to the accumulation of DNA damage that triggers apoptosis [130]. These results implicate Dicer involvement in DNA repair. Interestingly, it was shown in human and mouse cell lines, that inactivation of Dicer leads to inefficient DNA Damage Response (DDR) and the reduction in the level of Dicer-dependent small RNAs (ddRNAs), the regulatory RNAs involved in this process [137]. Another study reported that Dicer is involved in DNA double-strand break (DSB) repair [138], and DSB repair efficiency is reduced in *A. thaliana* in *DCL2*, *DCL3* and *DCL4* mutants [139]. Interestingly, it was demonstrated that hDicer is phosphorylated at position S1016 (the serine residue within the PPC cassette) upon induction of DSBs and recruited to facilitate DDR in the nucleus [140]. Nuclear Dicer was also implicated in the transcription-independent, global-genomic nucleotide excision repair pathway, in which it mediates the recruitment of a histone methyltransferase complex to the DNA damage site [141]. This way, Dicer presumably facilitates chromatin decondensation that, in consequence, allows repair factors to access the damage sites [142].

Dicer ribonuclease was found to be directly involved in the apoptosis process [143]. Apoptosis is a natural process of programmed cell death in multicellular organisms. One of the hallmarks of apoptosis is the fragmentation of chromosomal DNA leading to irreversible cell death. The process of apoptosis itself can be regulated by the Dicer-produced miRNAs [123]. Nevertheless, in a nematode *C. elegans* the apoptosis-induced caspases can cleave Dicer within the RNase IIIa domain, which results in releasing the C-terminal fragment of Dicer. Such truncated Dicer does not act as an RNase III enzyme; instead it displays the activity characteristic of DNases by initiating the chromosome fragmentation in the nucleus [143]. Such DNase activity has not been found for vertebrate Dicers; however, it was demonstrated that in human astrocytes, hypoxia-induced apoptosis is mediated by a decrease in hDicer levels via caspase-1 activation [144].

## 5. Conclusions

Domain fusions are a common occurrence throughout protein evolution [115]. In the case of Dicer-type proteins, the PAZ and RNase III domain fusion process has allowed to form the molecular rulers that can dice the dsRNA substrates into small RNAs of the defined length. The 21–23-nt products of Dicer cleavage seem to perfectly match Ago proteins, the miRNA/ siRNA-guided core effectors of RNA silencing. If the products are longer, instead of having a regulatory role, they would activate the intracellular RNA virus sensors (e.g., RIG-I helicases) which, in consequence, would induce the interferon pathway and trigger inflammation in vertebrate animals or activate antiviral response in lower organisms. On the other hand, shorter products could not provide a sufficiently specific mode of operation in terms of RNA–RNA interactions, as well as binding to specific proteins. Indeed, RNAs shorter than 20-nt were shown to bind inefficiently (or at all) to hDicer [145]. Thus, the “twentyish” seems a perfect RNA regulator.

Gain and loss of protein domains are important events that have been occurring abundantly during evolution of species. A minimal Dicer from *G. intestinalis* has only PAZ and two RNase III domains, which are entirely sufficient to specifically recognize and bind dsRNA and generate small RNAs of a certain length (~25-nt). Gaining the helicase domain has allowed Dicer-type proteins to serve a function of a cytosolic sensor of viral RNAs. However, the helicase domain of vertebrate Dicers seems not to be involved in the viral RNA recognition process. Instead, it provides another layer of regulation of substrate recognition by discriminating among pre-miRNA substrates, and between pre-miRNAs and pre-siRNAs. Consequently, gaining the helicase domain by ancestor Dicers has allowed to consolidate the cleavage, sensor and regulator activities in one protein [63]. Moreover, the acquisition of the helicase, DUF283 and dsRBD domains [81] has allowed Dicer proteins to develop binding platforms for interacting proteins. By choosing the protein partner, Dicer can control the specificity of substrate selection and cleavage [71,72,73], as well as the function performed in the cell [105,131]. Moreover, gaining the dsRBD domain equipped with NLS has allowed Dicer to shuttle between the cytoplasm and the nucleus upon specific signals [128,129]. Regarding the loss of domains, it can be observed that the Dicer proteins dedicated to miRNA production, e.g., insect Dicer-1 enzymes, have the degenerated helicase domain unable to hydrolyze ATP [25]. In addition, amazingly, removing of the N-terminal fragment of *C. elegans* Dicer by death-promoting caspases triggers the rearrangement of the remaining C-terminal fragment, containing one active RNsase III domain and one dsRBD domain. Such truncated *C. elegans* Dicer lacks the RNase III activity, but instead it benefits from being a DNase [143]. There is no doubt that Dicer-type enzymes have not yet revealed all their secrets; they are still an open book full of puzzles, brain teasers, and surprises; fortunately, we have the privilege to learn from this book.

## Abbreviations

ADAR	Double-stranded RNA-specific Adenosine Deaminase
Ago	Argonaute
bp	base pair
CAF	CARPEL FACTORY
cryo-EM	cryogenic Electron Microscopy
DCL	DICER-LIKE proteins
DDR	DNA Damage Response
ddRNA	Dicer-dependent small RNA
DSB	DNA Double-Strand Break
dsRBP	double-stranded RNA Binding Protein
dsRNA	double-stranded RNA
hDicer	human Dicer
HYL 1	Hyponastic leaves 1
miRNA	micro-RNA
NLS	Nuclear Localization Signal
NMR	Nuclear Magnetic Resonance
nt	nucleotide
OB fold	Oligonucleotide/Oligosaccharide-Binding fold
PACT	Protein Activator of the interferon-induced protein kinase
PPC	Platform-PAZ-Connector helix cassette
PPD	PAZ-Piwi Domain
pre-miRNA	miRNA precursor
pri-miRNA	miRNA primary precursor
RISC	RNA-induced Silencing Complex
SF2	Helicase Superfamily 2
siRNA	small interfering RNA
tasiRNA	trans-acting siRNA
TRBP	TAR RNA-binding protein
tRF	tRNA-derived Fragment
UTR	Untranslated Region
